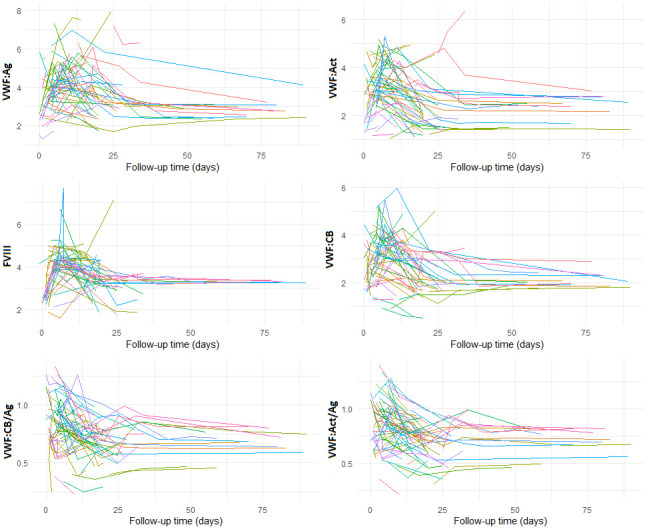# Early Postoperative Changes in Von Willebrand Factor Activity Are Associated With Future Bleeding and Stroke in HeartMate 3 Patients

**DOI:** 10.1097/MAT.0000000000002250

**Published:** 2024-06-19

**Authors:** Parsa Jahangiri, Kevin M. Veen, Iris van Moort, Jeroen H. Bunge, Alina Constantinescu, Jelena Sjatskig, Moniek de Maat, Jolanda Kluin, Frank Leebeek, Kadir Caliskan

**Affiliations:** From the * Department of Cardiology; † Cardiothoracic Surgery; ‡ Hematology; § Intensive Care, Erasmus MC-University Medical Centre Rotterdam, Rotterdam, The Netherlands.

**Keywords:** heart-assist devices/adverse effects, von Willebrand factor, hemorrhage/complications, heart failure/surgery, hemostasis, blood coagulation

## Abstract

Hemocompatibility-related adverse events (HRAEs), particularly gastrointestinal bleeding, remain a frequent complication after left ventricular assist device (LVAD) implantation. The current study sought to describe and analyze whether early (<60 days) postoperative von Willebrand factor (VWF) activity assays predict the risk of gastrointestinal bleeding and stroke. A prospective single-center study including 74 HeartMate 3 device recipients between 2016 and 2023 was undertaken. The postoperative trajectory of the VWF profile was analyzed using linear mixed-effect models and Cox models were used to quantify associations between an early postoperative dip (≤0.7) in VWF activity assay measurements and late outcomes. Preoperatively, the mean VWF:Activity (Act)/Antigen (Ag) and VWF:Collagen Binding (CB)/Ag ratios were 0.94 (95% confidence interval [CI] = 0.81–1.02) and 0.95 (95% CI = 0.80–1.03), respectively, decreasing to 0.66 (95% CI = 0.57–0.73) and 0.67 (95% CI = 0.58–0.74) within 40 days (*p* < 0.05). In patients with VWF:CB/Ag and VWF:Act/Ag ratios ≤0.7 significantly more gastrointestinal bleeding (hazard ratio [HR]: 2.53; 95% CI = 1.1–5.8, and HR: 3.7; 95% CI = 1.5–9.2, respectively) and hemorrhagic stroke events (HR: 3.5; 95% CI = 1.6–7.6 and HR: 4.9; 95% CI = 2.1–11.7, respectively) were observed throughout the entire late (>60 days) postoperative period. In patients with VWF:Act/Ag ratio ≤0.7 less ischemic stroke events were observed (HR: 0.11; 95% CI = 0.01–0.85). In conclusion, VWF:Act/Ag and VWF:CB/Ag ratios ≤0.7 in the early postoperative phase can be used as biomarkers to predict HRAEs during long-term LVAD support.

Continuous-flow left ventricular assist devices (CF-LVADs) have been increasingly used as a therapeutic option to provide cardiac support in selected patients with heart failure, both as bridge-to-transplant and, increasingly, as destination therapy.^1–3^ Regrettably, extended LVAD support entails possible hemocompatibility-related adverse events (HRAEs) like major bleeding, particularly gastrointestinal bleeding (GIB), and thromboembolic events which can affect quality of life and long-term survival for these patients.^2,3^ Bleeding is among the most common complications following LVAD implantation, with GIB affecting up to 30% of patients with CF-LVADS.^4^ Left ventricular assist device implantation results in an altered hemostatic balance as a consequence of acquired coagulation abnormalities due to blood–pump interactions, changes in hemodynamics, and the strict need for long-term anticoagulant treatment with oral anticoagulants and antiplatelet therapy.^5^ The multifactorial etiology of bleeding and thromboembolic events in this population complicates prediction of HRAEs.

Elevated shear stress within LVAD pumps can lead to abnormal cleavage of von Willebrand factor (VWF) multimers resulting in the loss of larger multimers, a hemostatic disorder known as acquired von Willebrand Syndrome (AvWS).^2,6^ Acquired von Willebrand Syndrome induced by LVADs has been highlighted as a possible cause of bleeding events, while concurrently demonstrating a potential lower risk of ischemic events.^7,8^ Hence, routine measurements of the VWF profile may aid in risk assessment. However, there remains a lack of information about the perioperative changes in VWF profile, the onset of AvWS in CF-LVADs patients and its correlation to late postoperative bleeding and ischemic events.

In the current study, we conducted an analysis of VWF profile parameters measured within the first 60 days following LVAD implantation. The aim of this study is to provide a detailed course of the early postoperative VWF profile and to evaluate the predictive capacity of early postoperative VWF measurements, particularly the VWF:Collagen Binding (CB)/Antigen (Ag) and VWF:Activity (Act)/Ag ratios, in relation to late bleeding and stroke events.

## Methods

### Study Design and Patient Population

A prospective single-center cohort study was performed at the Erasmus Medical Center including patients with a HeartMate 3. All HeartMate 3 patients between 2016 and 2023 in our institution with available VWF assays including VWF:Ag, VWF:Act, VWF:CB, and multimer analysis were included. As per our hospital protocol, all patients were required to undergo laboratory testing of VWF following LVAD implantation. Measurements of VWF:Act, VWF:Ag, VWF:CB, and factor VIII (FVIII) were routinely conducted preoperatively, immediately after surgery, and subsequently every 7 days for at least 5 weeks in HeartMate 3 patients. Additionally, to comprehensively capture the complete VWF profile during the initial 60 days, we collected data from unscheduled time points, in addition to routine measurements, within the first 60 days post-LVAD implantation. Approval was obtained from the institutional medical ethical committee to conduct this study (MEC-2017-1013).

### Antithrombotic Regimen

Antithrombotic regimen for all patients is warfarin (targeting an International Normalized Ratio (INR) within therapeutic range of 2.0–3.0) and aspirin 80 mg once a day. If a patient is aspirin intolerant, it is treated with clopidogrel. Time in therapeutic range was calculated based on INRs reported throughout the follow-up period.

### Von Willebrand Factor:Antigen and Activity Assays Assessment Methods and Multimer Analysis

Von Willebrand factor multimers, VWF:Ag, VWF:CB, and VWF:Act were measured following established methods from the literature.^9–11^ Von Willebrand factor:Ag was measured using polyclonal rabbit antihuman VWF antibody and horseradish peroxidase-conjugated antihuman VWF antibody in an enzyme-linked immunoassay. Von Willebrand factor:Act was measured with the Innovance VWF Ac reagent on a Sysmex CS 5100 using the manufacturer’s protocol. Von Willebrand factor:CB was measured with a chemiluminescent method performed on ACL AcuStar Analyzer. Von Willebrand factor multimers were measured with the Hydrasys 2 Scan instrument. We conducted densitometric analysis of VWF multimers according to the methodology outlined in our previously published study.^12^ The quantitative evaluation of VWF large multimers was performed using the “VWF large multimer index” as proposed by Tamura *et al.*^13^ Based on densitometric analysis, the VWF large multimer index was calculated as the ratio of the large multimer area of a patient to that of a control whose was analyzed in the next lane of the same VWF multimer analysis.^13^ Von Willebrand factor large multimer indices <80% indicate loss of VWF large multimers.^13^

### Outcome Definitions

Medical and laboratory data were derived from electronic patient records. We adopted the latest criteria for AvWS, which are based on laboratory assessments of normal or slightly decreased VWF:Ag levels, significantly reduced VWF activity assays, and abnormal VWF multimer electrophoresis.^14^ Gastrointestinal bleeding was defined as any suspicion or documented evidence of bleeding from the gastrointestinal tract, characterized by a decrease in hemoglobin levels and the presence of melena, hematochezia, hematemesis, or guaiac-positive stools.^15^ Ischemic stroke is defined as any focal neurologic deficit regardless of duration of symptoms with an associated infarct noted on neuroimaging.^16^ Intracerebral hemorrhage is defined as the initiation of neurologic dysfunction, validated by a positive cerebral computed tomography.^17^

### Endpoints

The primary endpoint was to assess whether VWF:Act/Ag and VWF:CB/Ag ratios ≤0.7 within the initial 60 days could predict HRAEs, including GIB and hemorrhagic stroke, during the entire course of LVAD support. Concurrently, an assessment was made to determine if patients with a ratio ≤0.7 exhibited a reduced risk of ischemic stroke. Von Willebrand factor:Act/Ag and VWF:CB/Ag reflect the function of the VWF protein, and values above 0.7 are considered within the normal range.^14,18^ Therefore, we have chosen to use 0.7 as the threshold when predicting HRAEs with these ratios. If a patient passed away within 60 days postoperatively, they were excluded from the study. The secondary endpoint entailed the statistical modeling of the postoperative trajectory during the initial 60 days for the following VWF profile parameters: VWF large multimers, VWF:Ag, VWF:Act, VWF:CB, VWF:Act/Ag, VWF:CB/Ag.

### Statistical Analysis

Continuous parameters were expressed as median and interquartile range (IQR) (non-Gaussian distribution) or as mean and 95% confidence interval (CI) (Gaussian distribution). Categorical parameters were expressed as number of patients and percentage. Comparisons among continuous variables between patient groups (normal *versus* abnormal VWF activity assay) were made with the one-way analysis of variance or the Kruskal–Wallis test, as appropriate. Categorical variables were analyzed using the χ^2^ test. Individual trajectory of VWF subunits were visualized using spaghetti plots. The statistical modeling of the overall VWF profile trajectory, aimed at handling data across all time points, was performed using a linear mixed-effects model. This model included random patient intercept and slope over time to account for higher correlation within repeated measurements. Flexibility over time was allowed using natural splines.^19^ The models were visualized using effect plots showing the modeled dependent outcome variable over time including its 95% CI.^20^ Two-tailed *p* values <0.05 were considered statistically significant. No correction for multiple testing was performed. Kaplan–Meier curves stratified by VWF cutoff groups were constructed to calculate and visualize the incidence of GIB and stroke in the postimplant follow-up period. The log-rank test was used to compare strata. Strata were defined as having an early dip under 0.7 of VWF activity ratio measurements, based on the patient data. The Cox proportional hazard model was used to determine the hazard ratios of predictors on GIB and other HRAEs with the early postoperative dip. We used Schoenfeld residuals to test the assumption of proportional hazards.^21^ When the assumption of proportional hazards did not hold, a time-varying model was used to derive hazard ratios over different periods of follow-up. Analyses were performed using statistical software R (version 4.1.2) using packages lme4, NLME, survival, survminer.

## Results

### Baseline Patient Characteristics

Table 1 provides a summary of the patient characteristics. A total of 113 patients underwent HeartMate 3 LVAD implantation between September 2016 and February 2023. Seventy-six (65%) patients had available VWF profile measurements. For the remaining patients, VWF measurements were unavailable because not all patients underwent protocol-specified laboratory testing for their VWF profiles, without specific patient selection. A total of 362 time points with VWF parameters were available. Seventy-four (97.4%) patients survived for at least 60 days and were therefore included. Clinical follow-up was performed until May 2023. Median age at surgery was 53.3 years (IQR: 40–68) and 51 (68.9%) patients were male. The median duration of follow-up was 26.8 months (IQR: 8–40). The total follow-up was 93 patient-years. Patients with an abnormal VWF activity ratio were observed to have a higher proportion of males and a lower percentage with a smoking history. Additionally, they exhibited higher LVAD speed and flow and lower right atrial pressure. The time spent in the therapeutic range for patients with abnormal VWF activity assays was 49% (IQR: 40–55), compared with 53% (IQR: 42–59) for patients with normal postoperative VWF activity assays (*p* > 0.05).

**Table 1.** Baseline Characteristics of HeartMate 3 Patients Stratified Against Normal or Abnormal VWF Activity Assay

Baseline Characteristics	Overall (n = 74)	VWF Activity Ratio <0.7 (n = 53)*	VWF Activity Ratio >0.7 (n = 21)	*p* Value
Demographics				
•Age (years)	53.3 (40.1–68.3)	53.5 (40.3–68.6)	52.8 (39.0–66.1)	ns
•Male	51 (68)	38 (72)	13 (62)	0.046
Body mass index (kg/m)	23.9 (19.8–25.8)	24.0 (20.0–25.9)	23.8 (19.7–25.5)	ns
Body surface area (m^2^)	1.97 (1.88–2.04)	1.96 (1.92–2.03)	1.99 (1.91–2.07)	ns
Systolic BP	100 (90–110)	105 (92–113)	98 (89–108)	ns
Diastolic BP	65 (57–72)	66 (57–72)	65 (56–71)	ns
Heart failure etiology				
Nonischemic	37 (49)	28 (53)	10 (48)	ns
Ischemic	38 (51)	25 (47)	11 (52)	ns
Comorbidities				
Diabetes mellitus	11 (16)	8 (21)	3 (14)	ns
Hypertension	8 (12)	6 (16)	2 (10)	ns
ICD therapy	43 (57)	30 (57)	13 (62)	ns
Neurologic event	6 (9)	5 (9)	1 (5)	ns
COPD	4 (5)	3 (6)	1 (5)	ns
Smoking history	40 (53)	28 (70)	12 (92)	0.019
INTERMACS patient profile				
Class 1	16 (22)	12 (23)	4 (19)	ns
Class 2	11 (15)	9 (15)	2 (14)	ns
Class 3	23 (31)	16 (30)	7 (33)	ns
Class ≥4	24 (32)	16 (30)	8 (38)	ns
TAPSE (mm)	14 (12–16)	15 (12–17)	14 (13–16)	ns
Temporary MCS before LVAD				
•IABP	7 (9)	4 (8)	3 (14)	ns
•Impella	6 (8)	4 (8)	2 (10)	ns
•VA-ECMO	8 (11)	5 (9)	3 (14)	ns
RA pressure (mm Hg)	10 (7–14)	9 (6–14)	10 (7–14)	0.044
Controller pump parameters				
RPM (r/min)	5,600 (5,400–5,800)	5,600 (5,500–5,900)	5,200 (5,100–5,400)	0.033
Flow (L/min)	4.4 (4.1–4.8)	4.6 (4.3–5.0)	4.1 (3.8–4.5)	0.022
PI	4.2 (3.8–5.3)	4.5 (4.1–5.8)	3.8 (3.3–4.3)	ns
Pulse power (W)	3.8 (3.8–4.0)	3.8 (3.8–4.0)	3.9 (3.8–4.1)	ns
Baseline laboratory data				
Creatinine (µmol/L)	107 (85–141)	110 (88–151)	101 (80–135)	ns
Hemoglobin (g/dl)	12.0 (10.5–13.7)	11.9 (10.1–13.6)	12.2 (10.5–13.9)	ns
Platelet count	217 (159–271)	220 (161–275)	215 (153–265)	ns
CRP (mg/L)	3 (0.9–9.3)	2.9 (0.8–9.1)	3.1 (1.0–9.5)	ns
INR	1.5 (1.22–1.77)	1.5 (1.21–1.78)	1.51 (1.21–1.76)	ns
PTT (s)	39 (30–47)	38 (29–46)	40 (32–49)	ns
Potassium	4.0 (3.6–4.3)	4.1 (3.7–4.4)	3.8 (3.5–4.2)	ns
Sodium	140 (132–145)	141 (133–146)	138 (131–143)	ns
NT-proBNP (pg/ml)	190 (139–390)	191 (145–396)	180 (134–373)	ns
ICU stay (days)	12 (5––24)	13 (6–25)	11 (4–23)	ns
Length of stay after implantation (days)	27 (21–37)	26 (20–36)	28 (22–39)	ns
Loop diuretics	68 (92)	49 (92)	19 (90)	ns

Due to uniformity, all continuous variables are depicted as median (interquartile range) and categorical variables are depicted as count (percentage).

* This includes patients with either or both VWF activity ratio assays <0.7 in at least one postoperative measurement.

BP, blood pressure; COPD, chronic obstructive pulmonary disease; CRP, C-reactive protein; INR, international normalizes ratio; IABP, intra-aortic balloon pump; ICD, implantable cardioverter-defibrillator; ICU, intensive care unit; INTERMACS, Interagency Registry for Mechanically Assisted Circulatory Support; LVAD, left ventricular assist device; MCS, mechanical circulatory support; ns, non-significant; NT-proBNP, N-terminal-proBrain natriuretic peptide; PI, pulsatility index; PTT, Protombine time; RA, right atrial; RPM, rotations per minute; TAPSE, tricuspid annular plane systolic excursion; VA-ECMO, veno-arterial extracorporeal membrane oxygenator; VWF, von Willebrand factor.

### Postoperative Course of the von Willebrand Factor Profile

**Figure 1.**
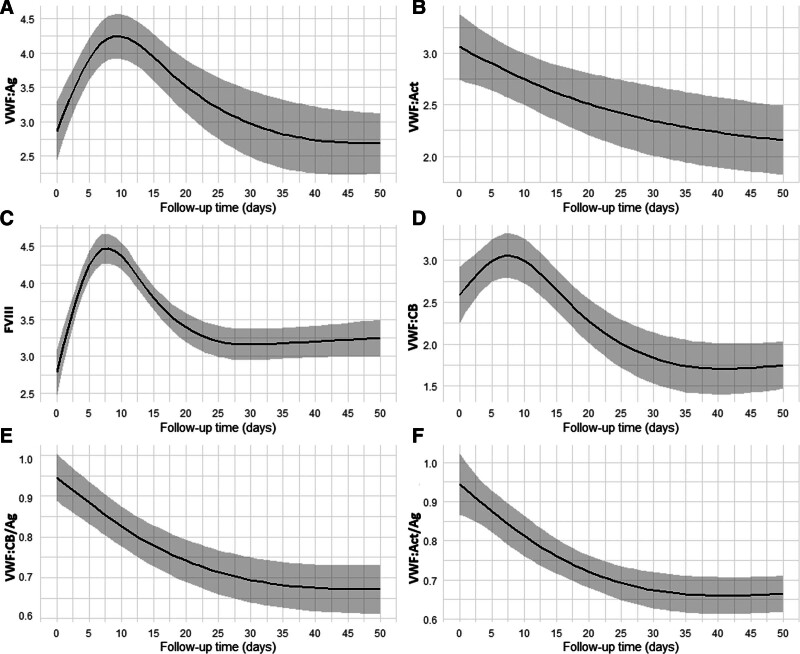
The modeled postoperative course of the VWF profile: (**A**) VWF:CB, (**B**) VWF:Act, (**C**) FVIII, (**D**) VWF:Ag, (**E**) VWF:CB/Ag ratio, and (**F**) VWF:Act/Ag. Estimated mean and 95% confidence intervals. Act, Activity; Ag, Antigen; CB, collagen binding; FVIII, Factor VIII; VWF, von Willebrand factor.

The modeled VWF:Ag, VWF:CB, and FVIII trajectory displayed an increase during the early postoperative phase (*p* > 0.05), followed by a significant decrease in the late postoperative phase, which reached a stable state approximately 40 days after the procedure (Figure 1, A–C). In contrast, the modeled trajectory of VWF:Act, VWF:CB/Ag, and the VWF:Act/Ag ratio showed a progressive decline immediately after surgery, with this trend persisting until also reaching a stable state around 40 days postoperatively (Figure 1, D–F). Von Willebrand factor:Ag value returned nearly to its baseline level, while both the VWF:Act/Ag and VWF:CB/Ag ratios declined below their respective baseline values after stabilizing. The modeled preoperative, baseline, mean value of the VWF:Act/Ag and VWF:CB/Ag ratio was 0.94 (95% CI = 0.81–1.02) and 0.95 (95% CI = 0.80–1.03), respectively. The modeled mean value of the VWF:Act/Ag and VWF:CB/Ag ratios after stabilization was 0.67 (95% CI = 0.58–0.74) and 0.66 (95% CI = 0.57–0.73), respectively. Patients experiencing GIB or hemorrhagic stroke events had a median VWF:Act/Ag ratio of 0.63 (IQR: 0.58–0.68) and VWF:CB/Ag ratio of 0.62 (IQR: 0.56–0.67), respectively, after stabilization. Nonbleeding patients had a median VWF:Act/Ag ratio of 0.74 (IQR: 0.68–0.79) and VWF:CB/Ag ratio of 0.75 (IQR: 0.69–0.80), respectively, after stabilization. Supplementary Figure, Supplemental Digital Content, http://links.lww.com/ASAIO/B295, illustrates spaghetti plots depicting the modeled trajectory of the individual VWF profile measurements. During the postoperative follow-up period (60 days), 50 patients (68%) had at least one measurement of the VWF:Act/Ag ratio falling ≤0.7, while 51 patients (69%) had the VWF:CB/Ag ratio drop ≤0.7 in at least one measurement. No patient who had dipped ≤0.7 in either the VWF:CB/Ag or VWF:Act/Ag ratio in one measurement subsequently showed levels exceeding 0.7 in a later follow-up measurement.

### Acquired von Willebrand Syndrome and Multimer Analysis

Multimer analyses were conducted solely for patients exhibiting abnormal VWF activity assays, albeit not systematically. A total of 31 (41.8%) patients had available multimer analyses. All patients with available multimer analyses exhibited severe loss of VWF large multimers (mean index of 48 ± 22% (normal index: 100)) within 30 days post-LVAD implantation. Given that the patients, as indicated above, also had normal VWF:Ag levels and significantly reduced VWF activity assays, all 31 patients met the diagnostic criteria for AvWS.

### Association Between von Willebrand Factor:Activity/Antigen and von Willebrand Factor:Collagen Binding/Antigen Dip and Gastrointestinal Bleeding Risk

**Figure 2.**
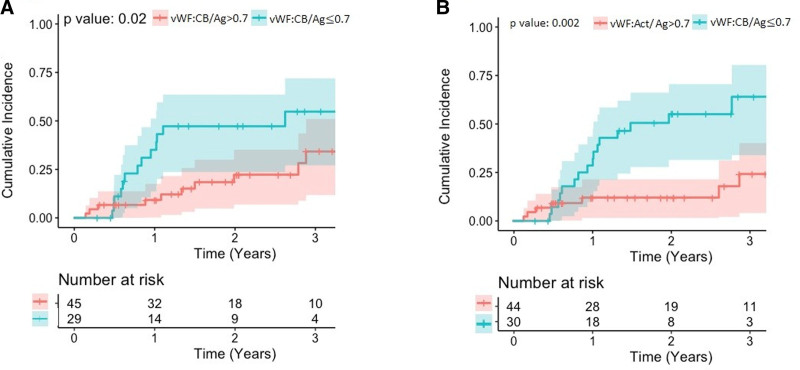
Kaplan–Meier curves for incidence of (**A**) gastrointestinal bleeding stratified by VWF:CB/Ag ratio and (**B**) gastrointestinal bleeding stratified by VWF:Act/Ag ratio. Act, Activity; Ag, Antigen; CB, collagen binding; VWF, von Willebrand factor.

In total, 24 of 74 patients (32%) experienced GIB during the long-term (>60 days) follow-up period. No patient had recurrence of bleeding, making all GIB events primary events. Gastrointestinal bleeding in our cohort had various causes: unknown in 11 patients (46%), arteriovenous malformation in five patients (21%), gastric ulcer in four patients (17%), gastritis in three patients (12.5%), and colonic ulcer in one patient (4%). The median day of first occurrence for GIB was 330 days (IQR: 130–590). Gastrointestinal bleeding risk after 60 days, stratified to VWF:Act/Ag ≤0.7 *versus* VWF:Act/Ag >0.7 and VWF:CB/Ag ≤0.7 *versus* VWF:CB/Ag >0.7 in the early postoperative phase (<60 days), is presented in Figure 2 and differed significantly between strata (*p* = 0.002 and *p* = 0.02, respectively). In patients with VWF:CB/Ag and VWF:Act/Ag ratios ≤0.7 observed in the early postoperative phase more GIB events (hazard ratio [HR]: 2.53; 95% CI = 1.1–5.8, *p* = 0.02 and HR: 3.7; 95% CI = 1.5–9.2, *p* = 0.002, respectively) were observed (Table 2).

**Table 2.** Results of Cox Proportional Hazards Models: Patients With a Dip in Either the VWF:Act/Ag or VWF:CB/Ag Ratio Under 0.7 *vs*. Patients With No Dip in the Early Postoperative Phase (<60 Days)

	Total n (%)	vWF:CB/Ag >0.7 n (%)	vWF:CB/Ag ≤0.7 n (%)	HRs (95% CI)	vWF:Act/Ag >0.7 n (%)	vWF:Act/Ag ≤0.7 n (%)	HRs (95% CI)
Adverse events							
GIB	24 (32.4)	5 (13.9)	19 (50)	2.53 (1.1–5.8)	4 (11.1)	20 (52.6)	3.7 (1.5–9.2)
Stroke							
Ischemic	8 (10.8)	5 (13.9)	3 (7.9)	0.29 (0.06–1.3)	6 (16.7)	2 (5.3)	0.11 (0.01–0.9)
Hemorrhagic	5 (6.8)	0	5 (13.2)	3.5 (1.6–7.6)	0	5 (13.2)	4.9 (2.1–11.7)
Death + hemorrhagic stroke + GIB	31 (41.9)	6 (19)	25 (65.6)	2.8 (1.4–5.5)	7 (19.4)	26 (68.4)	2.7 (1.3–5.3)

Act, Activity; Ag, Antigen; CB, collagen binding; CI, confidence interval; GIB, gastrointestinal bleeding; HRs, hazard ratios; VWF, von Willebrand factor.

### Association Between von Willebrand Factor:Activity/Antigen and von Willebrand Factor:Collagen Binding/Antigen and Stroke

**Figure 3.**
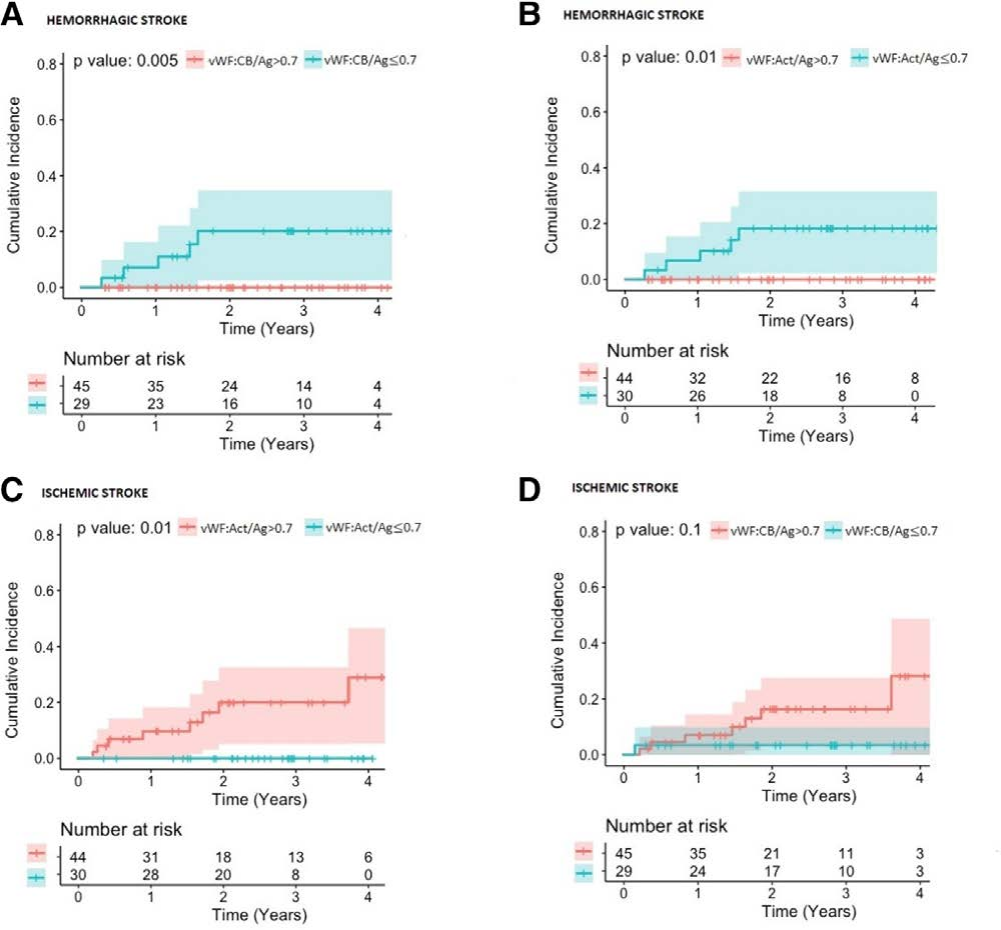
Kaplan–Meier curves for incidence of (**A**) hemorrhagic stroke stratified by VWF:CB/Ag ratio, (**B**) hemorrhagic stroke stratified by VWF:Act/Ag ratio, (**C**) ischemic stroke stratified by VWF:Act/Ag ratio, and (**D**) ischemic stroke stratified by VWF:CB/Ag ratio. Act, Activity; Ag, Antigen; CB, collagen binding; VWF, von Willebrand factor.

A total of 15 of 74 (20%) patients died during the long-term follow-up period. In total, ischemic stroke was recorded in eight of 74 patients, while hemorrhagic stroke occurred in five of 74 patients. Hemorrhagic transformation of the ischemic stroke occurred in no patients. Hemorrhagic and ischemic stroke risk after 60 days, stratified to VWF:Act/Ag ≤0.7 *versus* VWF:Act/Ag >0.7 and VWF:CB/Ag ≤0.7 *versus* VWF:CB/Ag >0.7 in the early postoperative phase (<60 days), is presented in Figure 3. Hemorrhagic stroke risk differed significantly in both the VWF:Act/Ag and VWF:CB/Ag strata (log-rank: *p* = 0.01 and 0.005, respectively) (Figure 3, A and B). Ischemic stroke differed significantly between both VWF:Act/Ag strata (Figure 3C) but not for the VWF:CB/Ag ratio strata (Figure 3D). In patients with VWF:Act/Ag ratios ≤0.7 observed in the early postoperative phase significantly less ischemic stroke events (HR: 0.11; 95% CI = 0.01–0.9, *p* = 0.02) were observed (Table 2).

## Discussion

Our prospective study characterized the early postoperative course (<60 days) of VWF profile parameters in HeartMate 3 patients and introduces a predictive marker, the VWF activity ratios, to aid in the prediction of HRAEs. We found that large VWF multimers, along with the VWF:CB/Ag and VWF:Act/Ag ratio exhibited a progressive postoperative decline and stabilized within 40 days postoperatively at values below baseline (*p* < 0.05). Acquired von Willebrand Syndrome was present in all patients of whom multimer analyses were available. In patients with VWF:CB/Ag and VWF:Act/Ag ratios ≤0.7 observed in the early postoperative phase, more GIB and hemorrhagic stroke events were observed during long-term LVAD support. In contrast, in patients with VWF:Act/Ag ratio≤0.7 less ischemic strokes were observed.

Our extensive laboratory data collection in a relatively large HeartMate 3 patient cohort allowed us to model the precise postoperative course of VWF profile parameters within the first 60 days post-LVAD implantation. Our data revealed an initial increase VWF:Ag levels during the first few weeks following LVAD implantation, followed by subsequent decreases and stabilization around baseline levels, consistent with previous studies.^7^ A similar pattern was observed in FVIII, as expected, because VWF acts as a protective carrier for FVIII, thereby preventing rapid proteolytic degradation.^22,23^ Consistent with current literature, since we observed that postoperative VWF:Ag values stabilized around baseline levels, we found that it is not the quantity of VWF that decreases after LVAD implantation but rather the VWF activity.^24,25^ However, little is known about the early postoperative trajectory of VWF activity. The reduction in VWF activity and VWF activity/antigen ratios begins immediately after LVAD implantation, persists for approximately 40 days postimplantation, and then stabilizes significantly below baseline levels for the remainder of our observation period. While our findings suggest a potential stabilization of the VWF profile, fluctuations in its parameters may persist over the long-term follow-up (>2 months). Previous studies have shown conflicting outcomes regarding the stability of the VWF profile in the long-term follow-up, with some indicating a progressive decline in VWF activity over time, while others suggesting stability and even potential recovery.^6,26^ Assuming, based on our findings, that VWF activity remains stable after 40 days, a measurement taken after this stabilization phase thus appears to be a valuable marker for predicting VWF activity throughout the entire long-term LVAD support period.

Bleeding diathesis due to reduced VWF activity, known as AvWS, is a recognized complication following LVAD implantation. Previous studies have revealed that a considerable number of patients develop AvWS shortly after the operation, despite the demonstrated greater preservation of high molecular weight multimers (HMWM) in HM3 patients compared to older generation LVADs.^1,8,24,25,27–29^ Our investigations regarding the reduction in VWF activity post-LVAD implantation, confirm the high prevalence of AvWS post-LVAD implantation. Because multimer analysis was not performed on all included patients, we were unable to determine the exact prevalence of AvWS in our HeartMate 3 cohort. Acquired von Willebrand Syndrome is primarily induced by elevated shear stress within LVAD pumps, which triggers abnormal cleavage of VWF multimers, consequently leading to the loss of larger multimers.^27^ High molecular weight multimers of VWF are particularly vital as they are highly effective in facilitating interactions with collagen and platelet receptors, thereby supporting primary hemostasis.^30^

Because AvWS has been highlighted as a possible cause of bleeding and ischemic events in LVAD patients, we evaluated the predictive capacity of early postoperative VWF measurements, particularly the VWF:CB/Ag and VWF:Act/Ag ratios, in relation to late bleeding and stroke events.^31^ While the literature suggests a correlation between the severity of AvWS and bleeding rates, VWF activity assays have received limited attention as potential predictors for bleeding or ischemic events.^25,28^ We found that in patients with VWF:CB/Ag and VWF:Act/Ag ratios ≤0.7 observed in the early postoperative phase (<60 days) more GIB and hemorrhagic stroke events were observed during long-term LVAD support. Meyer *et al.*^31^ previously attempted to use the VWF:CB/Ag ratio as a possible predictor for GIB but found no association. This disparity in results may be attributed to their comparison of patients with a ratio ≤0.8 against those above 0.8, whereas we used a cutoff of 0.7 according to the current guidelines.^14^ Values ≤0.7 are deemed abnormal, which may explain our observation of a higher GIB event rate among patients with a VWF:CB/Ag ratio ≤0.7 in comparison to Meyer *et al*.^14,18,31^ In contrast, patients with a dip ≤0.7 in the VWF:Act/Ag ratio exhibited a reduced risk of ischemic stroke. The observation that VWF activity ratios below 0.7 are associated with bleeding, while those above 0.7 are associated with ischemic events, highlights the clinical dilemma faced by clinicians in determining which VWF activity ratio level is preferable, as they strive to mitigate both major complications.

However, bleeding events in LVAD patients are multifactorial, involving platelet dysfunction, antiplatelet regimen, preimplant temporary mechanical circulatory support (MCS) and infection.^25,26,32,33^ All patients in our cohort were consistently treated with aspirin and oral warfarin. We observed no disparities in preimplant temporary MCS utilization between patients with pathologic and non-pathologic VWF activity ratios. While we emphasize the potential of VWF activity assays in predicting bleeding risk, it is essential to consider these assays alongside other pertinent variables when assessing bleeding risk for individual patients.

### Clinical Implications

Our data suggest that in LVAD recipients, a measured value ≤0.7 in the VWF:Act/Ag or VWF:CB/Ag ratios during the first 60 days post-LVAD implantation is a contributing factor to future bleeding events. While we conducted weekly measurements, this approach is less practical for clinical practice. Considering that patients who exhibit a VWF:Ag:/CB or VWF:Ag/Act ratio ≤0.7 before 40 days consistently maintain values below this threshold after 40 days, with no subsequent decrease, a singular postoperative measurement conducted after 40 days for both VWF activity ratios may serve as predictive markers for HRAEs, thereby alleviating the necessity for multiple assessments. Hence, our recommendation is to conduct a solitary measurement shortly after the 40 day stabilization phase to assess the risk of long-term bleeding events after LVAD implantation. Subsequently, based on these ratios, various clinical implications, particularly those concerning antithrombotic therapy, can be drawn. The dilemma regarding the antithrombotic regimen post-LVAD implantation revolves around striking a balance between preventing ischemic events while minimizing the risk of bleeding. Considering the protective effect of reduced VWF activity ratios against ischemic stroke and acknowledging the heightened risk of hemorrhagic and GIB in patients with low ratios, it may be prudent to reduce the intensity of antithrombotic regimens for people with low VWF activity ratios. However, given that bleeding and thromboembolic events in LVAD patients are multifactorial, decisions regarding antithrombotic regimens require a personalized approach. This involves a comprehensive assessment of various variables associated with LVAD-hemocompatibility, including VWF activity ratios, as well as specific patient characteristics.

### Strengths and Limitations

The strengths of our study are that we prospectively assessed VWF parameters in consecutive LVAD patients according to a standard protocol. We collected data of a large set of patients, all receiving a similar Heartmate 3 LVAD. This study has several limitations. Given the incomplete data available for some patients at different measurement time points, we used statistical methods to correct for missing data. Although we do not possess weekly data for all patients from the immediate postoperative period up to 60 days post-LVAD implantation, we have the most comprehensive dataset for the early postoperative phase following LVAD implantation. It would have been valuable to conduct functional assays at the time of bleeding events (GIB or hemorrhagic stroke). However, due to their significant cost and the fact that they are not yet standard practice, such assays were not performed. Due to the low event incidence, especially stroke, and the relatively small patient subset, we could not perform a multivariate Cox regression analysis.^34^ Thus, we conducted a univariate Cox regression analysis instead. The Siemens Innovance VWF:Act assay in our study measures the binding of VWF to platelet GPIb, albeit not under conditions of shear stress as the result of flow, thereby limiting its characterization as a fully functional test.^9^ However, currently the GPIb binding capacity remains one of the most reliable diagnostic markers for VWF activity.^9^

## Conclusions

Gastrointestinal bleeding and hemorrhagic stroke events occurred significantly more in patients with VWF:CB/Ag and VWF:Act/Ag ratios ≤0.7 observed in the early postoperative phase (<60 days). In patients with VWF:Act/Ag ratio ≤0.7 less ischemic strokes were observed. After following an initial decline which started immediately post-LVAD implantation, both ratios reached a stable state after approximately 40 days below their respective baseline level. This finding suggests that the use of a single postoperative measurement after 40 days of both VWF activity ratios can be used as predictive markers for HRAEs. Future prospective trials should explore whether postoperative VWF profile measurements could guide antithrombotic adjustments to reduce the risk of GIB and hemorrhagic and ischemic stroke.

## Supplementary Material

**Figure:**